# Prevalence of gastrointestinal parasitic infections in wild mammals of a safari park and a zoo in Bangladesh

**DOI:** 10.1002/vms3.1093

**Published:** 2023-02-06

**Authors:** Sabrina Ferdous, Joya Chowdhury, Tanjila Hasan, Pronesh Dutta, Md. Mizanur Rahman, Mohammad Mahmudul Hassan, Md. Rayhan Faruque, Mohammad Abdul Alim

**Affiliations:** ^1^ Department of Clinical Courses, Faculty of Veterinary and Animal Sciences Gono University Dhaka Bangladesh; ^2^ Department of Medicine and Surgery, Faculty of Veterinary Medicine Chattogram Veterinary and Animal Sciences University Chattogram Bangladesh; ^3^ Department of Physiology, Biochemistry and Pharmacology, Faculty of Veterinary Medicine Chattogram Veterinary and Animal Sciences University Chattogram Bangladesh; ^4^ Department of Pathology and Parasitology, Faculty of Veterinary Medicine Chattogram Veterinary and Animal Sciences University Chattogram Bangladesh

**Keywords:** Bangladesh, gastrointestinal parasitic infections, prevalence, safari park, wild mammals, zoo

## Abstract

In safari parks and zoos, wild animals are kept mainly for recreational purposes. Animals in these enclosures are also crucial for the education, research, and conservation aspect. To ensure better management and good health of wild animals in captivity, it is essential to monitor the occurrence of gastrointestinal (GI) parasitic (helminths and protozoa) infections. The current investigation was undertaken to investigate the prevalence of GI parasitic infections in wild mammals at Bangabandhu Sheikh Mujib (BSM) safari park and Chattogram (CTG) zoo of Bangladesh. A total of 72 individual faecal samples were collected from 25 species of wild mammals. Routine qualitative (e.g. direct smear, sedimentation, and flotation) and quantitative (e.g. McMaster technique) tests were performed to identify the eggs or oocysts of helminths and protozoa. Results demonstrated that wild mammals of both BSM safari park and CTG zoo were infected with a total of 17 genera/species of helminths and protozoa. The overall prevalence of GI parasitic infections in wild mammals of both zoological parks was 65.3% (95% confidence interval [CI]: 53.14–76.12), whereas it was 72.4% (95% CI: 52.76–87.27) in the BSM safari park and 60.5% (95% CI: 44.41–75.02) in the CTG zoo. In both zoological parks, infection with nematodes was more frequent compared to other helminth into the wild mammals. The herbivores were more infected with GI parasites than carnivores and omnivores of both BSM safari park and CTG zoo. The mean eggs/oocysts per gram of faeces was the highest in the carnivores compared to herbivores and omnivores of both enclosures. The findings of the current study demonstrated that wild mammals of both BSM safari park and CTG zoo suffered from various GI parasitic infections. Regular monitoring along with proper therapeutic measures may reduce the severe consequences of GI parasitic infections in captive wild animals.

## Graphical abstract

The study provides scientific evidence that gastrointestinal helminth and protozoan infections are very common in wild mammals of BSM safari park and CTG zoos in Bangladesh. Monitoring of these infections is very important for ensuring the better health of the animals.

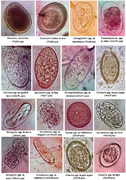

## 1 INTRODUCTION

Safari parks and zoos usually differ in their structure and principles. In these premises, wild animals are kept mainly for recreation and preservation of endangered species (Mir et al., 2016; Rahman et al., 2014). In safari parks, wild animals are allowed to roam freely in their specific territories, having a resemblance to their natural habitats. On the contrary, in zoos, animals are generally kept in enclosures that are entirely different from their natural habitats (Da Silva Barbosa et al., 2019). When wild animals are brought from wild to captivity (e.g. safari parks, zoos), the biology of those animals is altered due to the sudden exposure to an unfavourable and stressful environment. This kind of alteration increases the susceptibility to various infectious diseases (e.g. viral, bacterial, fungal, parasitic), and among those, gastrointestinal (GI) parasitic (helminths and protozoa) infections are the most common in captive wild animals (Adeniyi et al., 2015; Carrera‐Játiva et al., 2018; Kolapo & Jegede, 2017; Moudgil et al., 2015). In the natural habitat, animals might have a natural resistance against parasitic infections because of an ecological balance with their parasites. Furthermore, wild animals are less exposed to parasitic infections due to free‐roaming in ample areas and low density of animals (Da Silva Barbosa et al., 2019; Mir et al., 2016; Moudgil et al., 2015; Thawait et al., 2014). However, these infections induce negative impacts on the status, behaviour, reproduction, and survival of wild animals (Kvapil et al., 2017; Thawait et al., 2014).

An understanding of GI parasitic infections in captivity is vital for their documentation as these animals were brought from different countries and also collected from the local or wild territories. Furthermore, it is essential to know the mode of transmission and zoonotic potentiality of existing parasites within the wild animals of zoos and safari parks. In Bangladesh, there are two safari parks and six zoos. Unfortunately, there is no such extensive documentation of parasitic infections of wild mammals in these parks and zoos. Only a few studies have been conducted in zoos (e.g. Rangpur zoo, Bangladesh national zoo) and in Dulahazra safari park to identify the GI parasitic infections (Hossain et al., 2021; Khatun et al., 2014; Rahman et al., 2014; Raja et al., 2014). Therefore, the current investigation was designed to study the occurrence of GI parasitic infections in wild mammals of the most popular Bangabandhu Sheikh Mujib (BSM) safari park and Chattogram (CTG) zoo of Bangladesh.

## 2 MATERIALS AND METHODS

### 2.1 Ethical approval

All procedures were reviewed and approved by zoo and safari park authorities before conducting the research. The samples in this study were opportunisticall y collected from the territories or cages of wild mammals. Non‐invasive method was used during the collection of faecal samples.

### 2.2 Study periods and sites

The study was conducted on wild mammals of BSM safari park, Gazipur and Chattogram (CTG) zoo, Chattogram district of Bangladesh, between September 2018 and March 2019. The BSM safari park was established in 2003 and is the largest safari park in Bangladesh. It comprises a total of 15,418,523 m^2^ of open natural and closed enclosures. There were a total of 220 animal species including birds, reptiles, and 25 species of wild mammals that roam freely in their specified territories (Table S1). Deworming was practiced twice a year with broad‐spectrum anthelmintic drugs on a rotation basis. On the other hand, the CTG zoo is the smallest zoo occupying an area of 242,812 m^2^. It was established in 1988 in the Chattogram district of the country and has become the most popular zoo in Bangladesh. There were about 300 animals belonging to 53 species, including 25 species of wild mammals. The zoo was consisted of both semi‐open and closed enclosures. Herbivores were kept in open‐top enclosures, and all carnivores were kept in closed enclosures (Table S2). Zoo enclosures were cleaned regularly, and anthelmintics were provided two to three times in a year. In both parks, carnivores were fed with beef or chicken, having a daybreak per week; omnivores were fed mainly with fruits, vegetables, beef, eggs, bread, so forth, whereas herbivorous animals were regularly provided with grass, cereal grains, vegetables, wheat bran, gram, so forth (Tables S1 and S2). Animals were monitored by the animal caretakers and treated by veterinarians.

### 2.3 Study animals, sample collection, and preservation

A total of 72 faecal samples were collected from 25 species of wild mammals of both safari park and zoo (Tables S1 and S2). Among those, 29 samples were collected from the wild mammals of the safari park (20 herbivores, seven carnivores, and two omnivores) and 43 from the zoo (22 herbivores, seven carnivores, and 14 omnivores). Approximately 5–10 g of individual faecal samples were collected from each animal species. Samples from carnivores and omnivores were collected in fresh condition, before cleaning the cages in the early morning. Samples from herbivores, housed in open‐natural (the BSM safari park) and open‐top (the CTG zoo) enclosures, were collected soon after the defecation. Immediately after collection, samples were placed in dry, clean, and labelled plastic sample containers and preserved with 10% formalin. All the samples were then transported to the parasitology laboratory of the department of pathology and parasitology of Chattogram Veterinary and Animal Sciences University, Chattogram, Bangladesh. Samples were kept at 4°C until laboratory examination.

### 2.4 Examination of faecal samples

**FIGURE 1:**
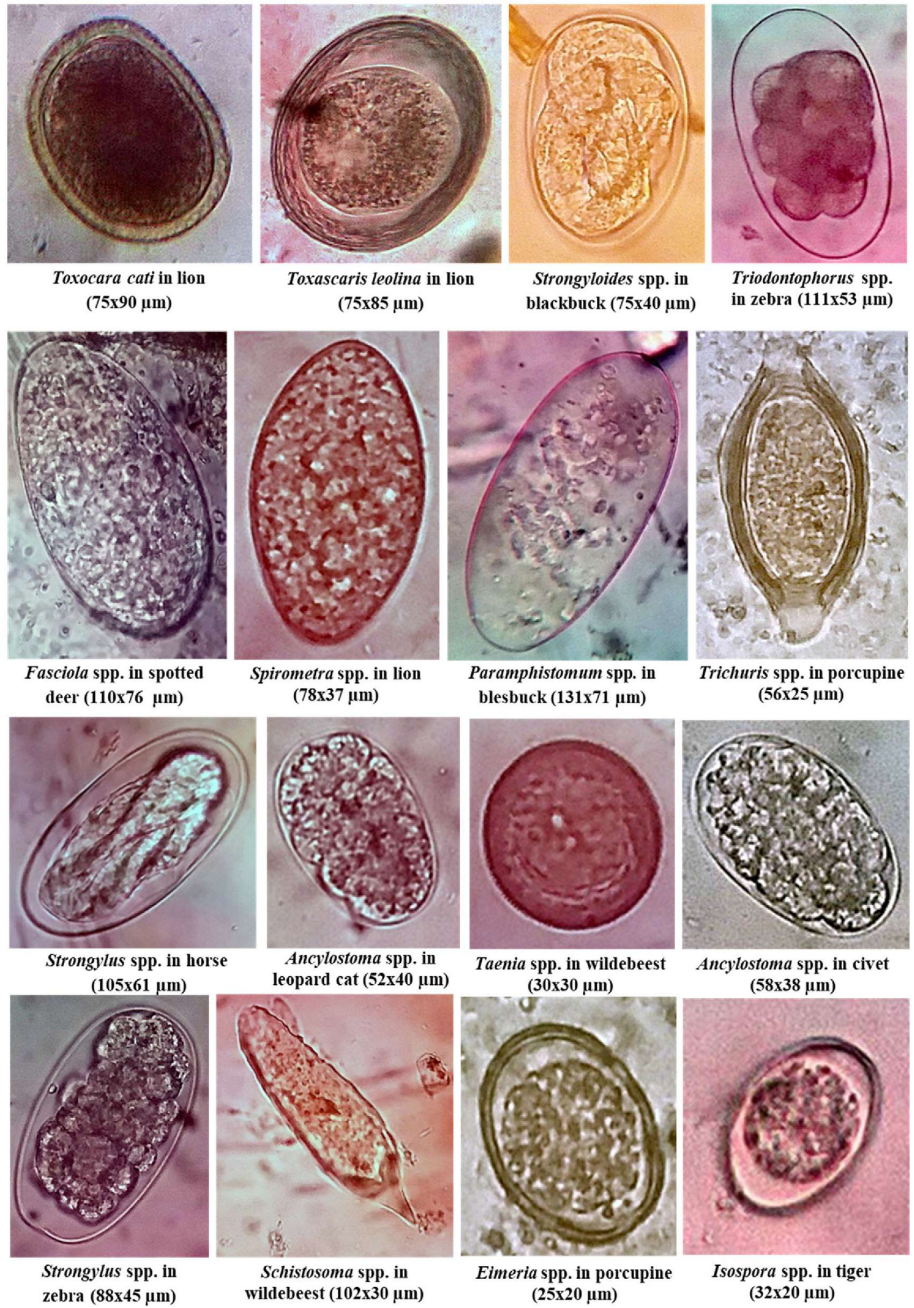
Eggs and oocysts of helminths and protozoa of wild mammals of the Bangabandhu Sheikh Mujib (BSM) safari park and Chattogram (CTG) zoo of Bangladesh.

Faecal samples were homogenized, and undigested faecal materials were removed through straining. Direct smears were prepared by taking a drop of faecal suspension on a glass slide. For the test tube floatation technique, approximately 3 g of faeces was thoroughly mixed with 50 ml of flotation fluid (saturated salt solution). The resulting faecal suspension was poured through a strainer to remove coarse faecal materials. The filtrate was then poured into a test tube and kept stand‐still for 15 min after placing a coverslip on top of each test tube touching the convex meniscus. The coverslip was lifted off and immediately placed on a glass slide for examination. For the simple sedimentation technique, the faecal suspension was kept stand‐still for about 15 min, and a drop of sediment was examined after discarding the supernatant. The intensity of infections was determined using the McMaster technique (Soulsby, 1982; Urquhart, 1996). Briefly, 4 g of faeces was mixed thoroughly with 56 ml of saturated salt solution. Then, both chambers of the McMaster slide were filled with the diluted suspension and allowed to stand for 5 min before microscopic examination. The eggs and oocysts were then counted. The morphological features of the eggs of helminths and oocysts of protozoa were identified using the published literature (Arjun et al., 2017; Berentsen et al., 2012; Rahman et al., 2014). Micrometry was performed to estimate the size of eggs and oocysts (Cable, 1950) (Figure 1).

### 2.5 Statistical analysis

The raw data were compiled, sorted in a spreadsheet using Microsoft Excel 2016, and then imported into STATA‐13^®^ (STATA Crop, 4905, Lake Way Drive, College Station, TX, USA) to conduct data analysis. Descriptive analysis with a 95% confidence interval (CI) was performed to estimate the prevalence of parasitic infections in animals based on their categories. Egg/oocyst per gram (EPG/OPG) of faeces was expressed by mean ± standard errors of the mean (SE). A chi‐square test was performed to calculate the presence of parasitic infections with associated risk factors in animal species. Analysis of variance was carried out to compare the parasitic load in faecal samples of animals. Results were considered significant when *p* ≤ 0.05.

## 3 RESULTS

### 3.1 Overall prevalence of helminth and protozoan infections in animals of both BSM safari park and CTG zoo

**FIGURE 2:**
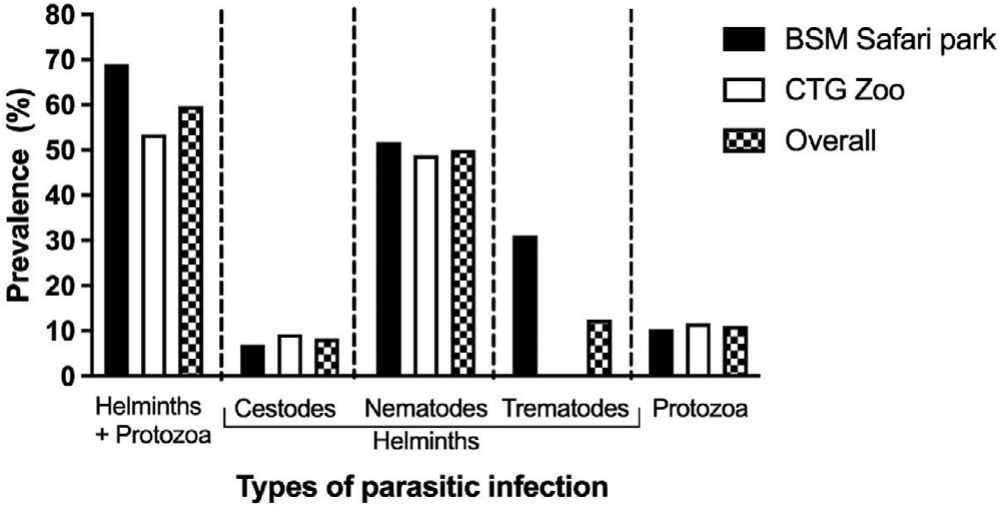
Overall prevalence of helminth and protozoan infections in animals of the Bangabandhu Sheikh Mujib (BSM) safari park and the Chattogram (CTG) zoo of Bangladesh.

**FIGURE 3:**
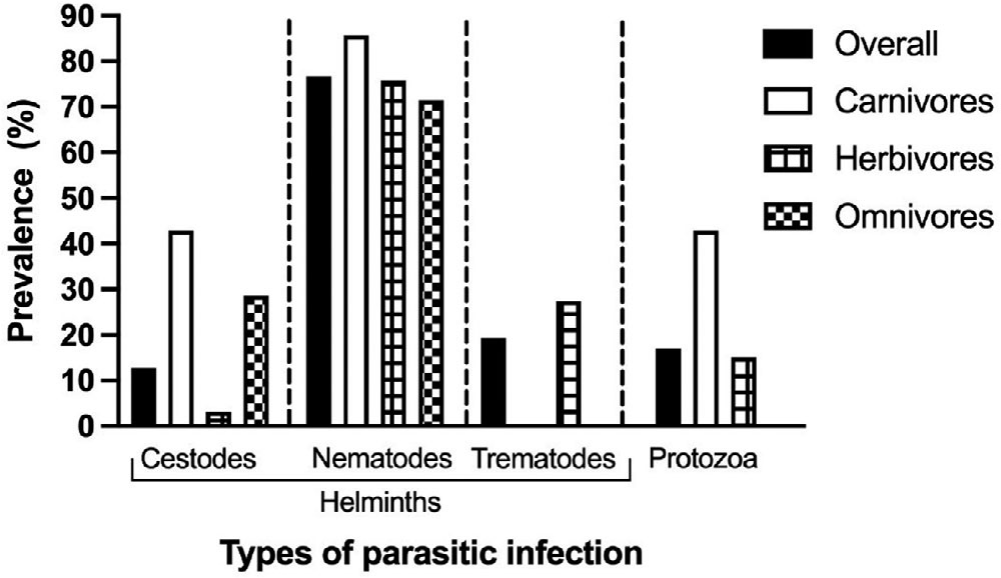
Overall prevalence of helminth and protozoan infections in different animals groups of the Bangabandhu Sheikh Mujib (BSM) safari park and Chattogram (CTG) zoo of Bangladesh.

The overall prevalence of GI parasitic infections (helminths and protozoa) was 65.3% (95% CI: 53.14–76.12, N = 72) in the wild mammals of both BSM safari park and CTG zoo. The overall helminths infections (91.5%; 95% CI: 79.62–97.63) prevailed over protozoan infections (17%; 95% CI: 7.65–30.81) in the wild mammals of the BSM safari park and the CTG zoo. Nematodes were the most prevalent helminths (76.6%; 95% CI: 61.97–87.7), followed by trematodes (19.2%) and cestodes (12.8%). Between the infection types, overall single (either of helminths or protozoa) infection (61.7%; 95% CI: 46.38–75.49) was higher than mixed (more than one helminths or protozoa) infections (38.3%; 95% CI: 24.51–53.62) in wild mammals of safari park and zoo (Figures 2 and 3).

### 3.2 Prevalence of gastrointestinal parasitic infections among the animal groups of both BSM safari park and CTG zoo

Among the animal groups, overall parasitic infections of helminths and protozoa were recorded highest in herbivores (78.6%; 95% CI: 63.19–89.7), followed by carnivores (50%; 95% CI: 23.04–76.96) and omnivores (43.7%; 95% CI: 19.75–70.12) (Table 1). The mixed infections of both helminths and protozoa were prominent in carnivores (71.5%; 95% CI: 29.04–96.33), whereas the single infection was predominant in omnivores (71.4%; 95% CI: 29.04–96.33). Among the identified parasites, helminths were more prevalent than the protozoa (*p* = 0.09) in all animal groups. The presence of protozoa was higher in carnivores (42.9%; 95% CI: 9.9–81.59), whereas helminths were higher in omnivores (100%; 95% CI: 59.04–100) (Table 1). Among the helminth parasites, nematodes (85.7%) and cestodes (42.9%, *p* = 0.007) were detected higher in carnivores than the other animal groups. Trematodes were detected only in herbivores (Table 1).

**TABLE 1:** Overall prevalence of helminth and protozoan infections in different animal groups of wild mammals of both Bangabandhu Sheikh Mujib (BSM) safari park and Chattogram (CTG) zoo of Bangladesh.

		Carnivores (N = 14)		Herbivores (N = 42)		Omnivores (N = 16)		
Variables	Categories	Prevalence (*n*)	95% CI	Prevalence (*n*)	95% CI	Prevalence (*n*)	95% CI	*p*‐Value
Presence of parasites	Yes	50 (7)	23.04–76.96	78.6 (33)	63.19–89.7	43.7 (7)	19.75–70.12	**0.018**
	No	50 (7)	23.04–76.96	21.4 (9)	10.29–36.81	56.2 (9)	29.88–80.25	
Types of infections	Single	28.6 (2)	3.67–70.96	66.7 (22)	48.17–82.04	71.4 (5)	29.04–96.33	0.144
	Mixed	71.4 (5)	29.04–96.33	33.3 (11)	17.96–51.83	28.6 (2)	3.67–70.96	
Types of parasites	Helminths	85.7 (6)	42.13–99.64	90.9 (30)	75.67–98.08	100 (7)	59.04–100	0.617
	Protozoa	42.9 (3)	9.9–81.59	15.1 (5)	5.11–31.9	0	0–40.96	0.09
Types of helminths	Cestodes	42.9 (3)	9.9–81.59	3 (1)	0.8–15.8	28.6 (2)	3.7–70.96	**0.007**
	Nematodes	85.7 (6)	42.13–99.64	75.8 (25)	57.74–88.81	71.4 (5)	29.04–96.33	0.802
	Trematodes	0	0–40.96	27.3 (9)	13.3–45.52	0	0–40.96	0.094

Abbreviation: CI, confidence interval.

### 3.3 Prevalence of gastrointestinal parasitic infections in the BSM safari park and CTG zoo

Between the two study sites, the overall prevalenc of GI parasitic infections was higher in the BSM safari park (72.4%; 95% CI: 52.76–87.27) than the CTG zoo (60.5%; 95% CI: 44.41–75.02) (Table 2). Among the positive samples, the mixed infection was prominant in the BSM safari park (42.9%; 95% CI: 21.82–65.98) than the CTG zoo (34.6%). Helminths were detected comaparatively higher in the animals of the BSM safari park (95.2%; 95% CI: 76.18–99.88) than the animals of the CTG zoo (88.5%; 95% CI: 69.84–97.55) (Table 2), whereas the occurrence was opposite in the case of protozoa. Among the helminths, nematodes and cestodes were detected higher in the animals of the CTG zoo (80.8% vs. 15.4%) than the animals of the safari park (71.4% vs. 9.5%), whereas the occurrence of trematodes (42.9%) was found only in the BSM safari park (Table 2).

**TABLE 2:** Overall prevalence of helminth and protozoan infections in wild mammals of the Bangabandhu Sheikh Mujib (BSM) safari park and Chattogram (CTG) zoo of Bangladesh.

		BSM safari park (N = 29)		CTG zoo (N = 43)		
Variables	Categories	Prevalence (*n*)	95% CI	Prevalence (*n*)	95% CI	*p*‐Value
Presence of parasites	Yes	72.4 (21)	52.76–87.27	60.5 (26)	44.41–75.02	0.296
	No	27.6 (8)	12.73–47.24	39.5 (17)	24. 98–55.59	
Types of infections	Single	57.1 (12)	34.02–78.18	65.4 (17)	44.33–82.79	0.563
	Mixed	42.9 (9)	21.82–65.98	34.6 (9)	17.21–55.67	
Types of parasites	Helminths	95.2 (20)	76.18–99.88	88.5 (23)	69.84–97.55	0.408
	Protozoa	14.3 (3)	3.05–36.34	19.2 (5)	6.55–39.35	0.654
Types of helminths	Cestodes	9.5 (2)	1.17–30.38	15.4 (4)	4.36–34.87	0.549
	Nematodes	71.4 (15)	47.82–88.72	80.8 (21)	60.65–93.44	0.452
	Trematodes	42.9 (9)	21.82–65.98	0	0–13.23	**0.000**

Abbreviation: CI, confidence interval.

### 3.4 Prevalence of helminth and protozoan infections in wild mammals of the BSM safari park and CTG zoo

A total of 17 genera of helminths and protozoa were identified in the samples of wild mammals. Among the positive samples, *Strongylus* spp. (38.3%) was the most frequent parasites in herbivores, followed by *Strongyloides* spp. (23.4%), *Fasciola* spp. (17%), and *Ancylostoma* spp. (12.8%) (Table 3). Accroding to locations, the presence of *Strongylus* spp. was almost similar in the animals of the both zoological parks. *Strongyloides* spp., *T. cati*, and *Toxascaris leolina* were higher in mammals of the CTG zoo than the BSM safari park. The occurrence of *Oesophagostomum* spp. and *Trichostrongylus* spp. was found only in the animals of the BSM safari park, whereas *Ancylostoma* spp., *Triodontophorus* spp., and *Trichuris* spp. were found in the CTG zoo (Table 3). We also observed the presence of trematodes only in the animals of the BSM safari park where the occurrence of *Fasciola* spp. (38.1%) was notable than other trematodes (Table 3). Between the detected cestodes in wild animals, *Spirometra* spp. (15.4%) was the most frequent in the animals of the CTG zoo than the BSM safari park. Between the two identified protozoan parasites, *Isospora* spp. (14.3%) was detected only in the animals of the BSM safari park, whereas *Eimeria* spp. (19.2%) infection was recorded in the animals of CTG zoo (Table 3).

**TABLE 3:** Prevalence of helminth and protozoan infections in wild mammals of the Bangabandhu Sheikh Mujib (BSM) safari park and Chattogram (CTG) zoo of Bangladesh.

		Prevalence percentage (positive samples)		
Helminths and protozoa	Infected animal species	BSM safari park (21)	CTG zoo (26)	Overall (47)
Cestodes				
*Spirometra* spp.	Fishing cat, leopard cat, lion, Bengal fox	4.8 (1)	15.4 (4)	10.6 (5)
*Taenia* spp.	Wildebeest	4.8 (1)	0	2.1 (1)
Nematodes				
*Toxocara cati*	Lion, tiger, jungle cat, fishing cat	9.5 (2)	11.5 (3)	10.6 (5)
*Toxascaris leolina*	Lion, jungle cat	4.8 (1)	7.7 (2)	6.4 (3)
*Strongyloides* spp.	Large Indian civet jungle cat, blackbuck, wildebeest, zebra	19 (4)	26.9 (7)	23.4 (11)
*Strongylus* spp.	Zebra, horse, pony, giraffe, elephant	38.1 (8)	38.5 (10)	38.3 (18)
*Ancylostoma* spp.	Leopard cat, large Indian civet, gibbon, rhesus macaque	0	23.1 (6)	12.8 (6)
*Triodontophorus* spp.	Zebra	0	3.8 (1)	2.1 (1)
*Trichuris* spp.	Indian crested porcupine	0	7.7 (2)	4.3 (2)
*Oesophagostomum* spp.	Giraffe	4.8 (1)	0	2.1 (1)
*Trichostrongylus* spp.	Giraffe	9.5 (2)	0	4.3 (2)
Trematodes				
*Fasciola* spp.	Gayal, zebra, nyala, wildebeest, kangaroo, spotted deer	38.1 (8)	0	17 (8)
*Paramphistomum* spp.	Gaur, blesbuk	9.5 (2)	0	4.3 (2)
*Schistosoma* spp.	Wildebeest, nyala	9.5 (2)	0	4.3 (2)
*Dicrocoelium* spp.	Wildebeest	4.8 (1)	0	2.1 (1)
Protozoa				
*Isospora* spp.	Tiger, lion	14.3 (3)	0	6.4 (3)
*Eimeria* spp.	Indian crested porcupine, gayal	0	19.2 (5)	10.6 (5)

### 3.5 Gastrointestinal helminth and protozoan infections load in wild mammals of the BSM safari park and CTG zoo

The mean EPG/OPG was measured in animal groups though there were some single samples and the number of the positive cases was one or two. The mean EPG/OPG of GI parasites in the wild mammals of both BSM safari park and CTG zoo was not statistically significant (5385.7 ± 4741.4 and 5326.9 ± 3416.3, respectively) (Tables S3 and S4). The mean EPG/OPG was higher in carnivores (19,785.7 ± 13978.6) followed by herbivores (3124.2 ± 2588.46), and omnivores (1428.6 ± 620.9) (Tables S3 and S4). Between the locations, the mean EPG/OPG in herbivores was higher in the CTG zoo (6466.7 ± 21982.68) than the BSM safari park (338.9 ± 403.13), but it was completely opposite in the carnivores where it was higher in the animals of BSM safari park (35,666.7 ± 55,811.95) than in the animals of the CTG zoo (7875 ± 14751.58) (Tables S3 and S4).

## 4 DISCUSSION

Gastrointestinal (GI) parasitic infections have adverse effects on the survival of wild animals and their welfare. It is very important to monitor the GI parasitism (helminths and protozoa) to ensure a better management and good health of animals in captivity. The occurrence of GI parasitic infections has been reported in various zoos and national parks of the world (Lim et al., 2008; Mir et al., 2016; Maske et al., 1990; Opara et al., 2010; Parsani et al., 2001; Rahman et al., 2014; Raja et al., 2014; Singh et al., 2006; Thawait et al., 2014). The current investigation has documented the occurrence of GI parasitic infections in BSM safari park and CTG zoo. The overall prevalence of GI parasitic infections in wild mammals of safari park and zoo was consistent with previously published reports (Da Silva Barbosa et al., 2019; Kolapo & Jegede, 2017). Higher (71%–77%) (Cordón et al., 2008; Holsback et al., 2013; Opara et al., 2010; Rahman et al., 2014) and lower prevalences (40%–60%) (Chakraborty et al., 2016; Lim et al., 2008; Parsani et al., 2001; Thawait et al., 2014) of GI parasitic infections were also recorded in wild mammals in captivity. These differences might be due to the variations in geography, captivity conditions, feed and feeding management, sources of water, use of anthelmintics, and husbandry practices (Fagiolini et al., 2010). In the BSM safari park, most of the animals (e.g. herbivores) shared the same habitat, and it was hard to maintain the effective anthelmintic dose for each individual, and this leads to higher occurrence of GI parasites. In addition, active transmission may also takes place in wild or semi‐wild habitats, and regular cleaning and dung removal may not possible on a regular basis. Environmental contamination facilitates the transmission of different stages of parasites through contaminated water or fodder, and even mechanically with the zoo workers (Adetunji, 2014; Mir et al., 2016; Otegbade & Morenikeji, 2014).

Helminth infections were more common than protozoan infections in the animals of both study sites, which was consistent with the prior studies (Adeniyi et al., 2015; Lim et al., 2008; Mir et al., 2016; Opara et al., 2010; Rahman et al., 2014; Raja et al., 2014). This finding suggested that the nature of the life cycle of helminths facilitates their survival in the parks or zoos, contributing to more helminth infections. Higher helminth infections in safari parks than in the zoo might be due to the favourable climatic conditions that favour the transmission and survival of helminths in the areas (Rahman et al., 2014). In contrast, protozoan infections were higher in zoo animals, which can be explained by the favourable factors required for the completion of life cycle, lower infective doses, and ease of transmission in zoo environment (Cordón et al., 2008; Levecke et al., 2007). Among the helminths, the prevalence of infections with nematodes was higher than other types of helminths in both sites, and this was accordant with previous studies (Rahman et al., 2014; Shemshadi et al., 2015). Higher occurrence of infections caused by nematodes was because of their direct life cycle with no involvement of intermediate hosts and being able to be transmitted through contaminated feed, water, and soil (Mir et al., 2016). On the other hand, trematodes and most of the cestodes require at least one intermediate host to complete their life cycle and for potential transmission. This might be the reason for lower occurrence of trematodes and cestodes infections in all animal groups of both study sites (Atanaskova et al., 2011; Mir et al., 2016).

Of all nematodes, *Strongylus* spp. had a higher occurrence in herbivores, which was consistent with previous studies (Aviruppola et al., 2016; Garijo et al., 2004; Rahman et al., 2014). This could be explained by its proliferative life cycle and survival capacity in the harsh environment (Aviruppola et al., 2016; Rahman et al., 2014). In both study sites, all the samples were collected from zebra, and horses were found infected by helminths, which indicated the active transmission of the parasite in grouped animals. *Strongylus* spp. have already been reported in most herbivores, for example, horses (Hinney et al., 2011; Umar et al., 2013; Uslu & Guclu, 2007), zebras (Fagiolini et al., 2010; Wambwa et al., 2004), giraffes (Nosal et al., 2016; Varadharajan & Pythal, 1999), and elephants (Fagiolini et al., 2010; Shahi & Gairhe, 2019; Varadharajan & Pythal, 1999). The presence of *Strongyloides* spp. in zebras, wildebeests, blackbucks, and blesbucks was also reported in previous studies (Mir et al., 2016; Varadharajan & Pythal, 1999). *Oesophagostomum* spp. and *Trichostrongylus* spp. were found in giraffes, and this was considered the first report from Bangladesh. Both parasites have been reported most commonly in ruminants and wild herbivores, which might get the infections through grazing in the same pasture along with other livestock of the safari park (Abuessailla et al., 2011; Farooq et al., 2012). *Triodontophorus* spp. in zebras were reported by prior studies, and these studies stated that the parasite has a higher prevalence in free‐ranging zebras (Wambwa et al., 2004). *Trichuris* spp. was found in captive porcupines and was not reported in Bangladesh before. The presence of *Trichuris* spp. in bristle‐spined porcupines was reported by Kuniy and Brasileiro (2006). Among carnivores, the presence of *T. cati* and *T. leolina* in jungle cats of the zoo and lions of both sites was consistent with previous studies (Acharjyo, 2004; Adeniyi et al., 2015; Fagiolini et al., 2010; Raja et al., 2014; Tabaripour et al., 2018). Carnivores usually get these infections through ingesting the intermediate hosts directly (Sepalage & Rajakaruna, 2020). Some literature indicated the co‐occurrence of both *Toxocara* spp. and *T. leonina* in canids and felids (Dalimi et al., 2006; Labarthe et al., 2004) and suggested that the occurrence is highly variable and depends on factors such as seasonal variability, age of an individual, climate change, and environmental conditions (Okulewicz et al., 2012). The presence of *T. cati* in fishing cats, *Strongyloides* spp. in jungle cats, and *Ancylostoma* spp. in leopard cats reported previously in several studies (Acharjyo, 2004; Moudgil et al., 2015). *Ancylostoma* spp. was also detected from gibbons, rhesus macaques, and from large Indian civets of the CTG zoo, and this was also reported by previous studies (Adedokun et al., 2002; Habtamu et al., 2017; Pourrut et al., 2011). Infections by *Ancylostoma* spp. may occur when infective larval stages are ingested directly by the host or through penetrating the skin (percutaneous route) while coming in direct contact with contaminated soil (Colon & Patton, 2012).

This study found a higher occurrence of *Fasciola* spp. infections in herbivores, which was consistent with previous studies (Khatun et al., 2014; Rahman et al., 2014). The occurrence of *Fasciola* spp. infections in the animals of the BSM safari park was suspected to be connected with the intermediate host (e.g. mud snails) that was observed near the water bodies. The herbivores that were infected by *Fasciola* spp. (e.g. nyalas, zebras, wildebeests, spotted deers, and gayals) shared the same habitats that might facilitate the cross‐transmission of parasites’ eggs. Trematodes found in gayals, blesbucks, nyalas, and wildebeests in our study were supported by findings of the previous studies (Boomker et al., 1991; Rahman et al., 2014). Two species of cestodes were identified, and all *Spirometra* spp. were more frequently identified from carnivores. *Spirometra* spp. in lions, jungle cats, and leopard cats in our study was consistent with the findings of previous studies (Berentsen et al., 2012; Khatun et al., 2014; Lim et al., 2008; Moudgil et al., 2015; Raja et al., 2014). Though there was no record of finding *Spirometra* spp. in Bengal foxes, Scioscia et al. (2014) reported the first finding of *Spirometra erinacei* in Pampas fox as a new definitive host.

Among the protozoa, *Isospora* spp. and *Eimeria* spp. infections were found in carnivores (lions and tigers) of the BSM safari park and herbivores (gayals and porcupines) of the CTG zoo. *Isospora* spp. in tigers and lions were reported by several prior studies (Berentsen et al., 2012; Bjork et al., 2000; Chauhan et al., 1973; Fagiolini et al., 2010; González et al., 2007; Mahali et al., 2010; Muraleedharan & Iswaraiah, 1984). Tigers or lions might get the infections by ingesting the infective oocysts from the environment or through an intermediate host that contains the infective tissue stage of the parasite (Colon & Patton, 2012).

Some of the helminths isolated from wild mammals such as *Trichiuris* spp., *T. cati*, *Ancylostoma* spp., *Strongyloides* spp., *Fasciola* spp., *Schistosoma* spp., and *Taenia* spp. are also important for public health (Mohd‐Shaharuddin et al., 2019; Youn, 2009). In human, *Trichiuris* spp. infect intestinal mucosa, and this leads to various symptoms such as bloody diarrhoea, typhlitis, and colitis (Cooper et al., 2013; Xie et al., 2018). *Toxocara cati* causes visceral larva migrans, ocular larva migrans, and covert toxocariasis, whereas *Ancylostoma* spp. causes cutaneous larva migrans (Marques et al., 2012; Overgaauw and van Knapen, 2013). *Strongyloides* spp. has also been reported for cutaneous, intestinal, pulmonary, or disseminated human infections (Fagiolini et al., 2010; Jaleta et al., 2017). *Schistosoma* spp. causes various diseases in humans including inflammatory and obstructive disease in the urinary system (*Schistosoma haematobium*) or liver fibrosis, hepatosplenic, and intestinal infections (*Schistosoma mansoni* and *Schistosoma japonicum*) (Colley et al., 2014; Gryseels et al., 2006; Kebede et al., 2020). In this study, we did not examine the samples from the animals caretakers which might determine and confirm the potential cross‐transmission of parasites to human population. This limitation could be addressed by future studies.

## 5 CONCLUSIONS

This study provides the scientific evidence that gastrointestinal helminths and protozoan infections are very common in wild mammals of the BSM safari park and CTG zoos in Bangladesh. Monitoring of these infections is very important for ensuring better health of animals. This is also important from public health aspects. As animal caretakers are in close contact with wild mammals, there are risks of cross‐transmission of infections. Furthermore, these animals may act as reservoir hosts for many of such zoonotic parasites which may ultimately result in active transmission to the human population. We recommend further detailed molecular and epidemiological investigation on the occurrence of parasitic infections in the animals of safari parks and zoos in Bangladesh with the identification of their associated risk factors.

## AUTHOR CONTRIBUTIONS

*Investigation, methodology, formal analysis, resources visualization, writing–original draft, and writing–review and editing*: Sabrina Ferdous. *Investigation, methodology, and visualization*: Joya Chowdhury. *Investigation, methodology, and validation*: Tanjia Hasan. *Data curation, formal analysis, software, and validation*: Pronesh Dutta. *Data curation, formal analysis, software, validation, visualization, and writing–review and editing*: Mohammad Mahmudul Hassan. *Conceptualization, investigation, methodology, and supervision*: Md. Mizanur Rahman. *Data curation, formal analysis, software, validation, visualization, and writing–review and editing*: Md. Rayhan Faruque. *Conceptualization, funding acquisition, investigation methodology, project administration, resources, supervision, validation, visualization, writing–original draft, and writing–review and editing*: Md. Abdul Alim.

## CONFLICT OF INTEREST

The authors declare no conflict of interest.

### ETHICS STATEMENT

All procedures were reviewed and approved by zoo and safari park authorities before conducting the research. The samples in this study were opportunistically collected from the territories or cages of wild mammals. No invasive method was used during the collection of feacal samples.

### PEER REVIEW

The peer review history for this article is available at https://publons.com/publon/10.1002/vms3.1093.

## Supporting information

Table S1

Table S2

Table S3

Table S4

## Data Availability

Data files associating this research are available at https://figshare.com/s/1d0c620beeb2a0e38f97.
