# Supplementary material for: Prevalence of gastrointestinal parasitic infections in wild mammals of a safari park and a zoo in Bangladesh
Source: Vet Med Sci. 2023 Feb 6;9(3):1385–94. doi: 10.1002/vms3.1093 (PMC10188080; doi:10.1002/vms3.1093)
Supplement: Supplementary file 1 — Table S1 [file VMS3-9-1385-s003.docx]

**Supplementary table 1: Sample collection from animal species of BSM safari park of Bangladesh with their habitat types and major feed ingredients**

| **Sl. No.** | **Animal species** | **Number of Samples** | **Nature of enclosures** | **Major feed ingredients** |
| --- | --- | --- | --- | --- |
| **Herbivores** | | | | |
|  | Elephant  (*Elephas maximus*) | 5 | Open natural grassland and cemented feeding shed | Banana tree, carrot, sugar cane, grass, pumpkin, tomatoes |
|  | Pony  (*Equus ferus caballus*) | 2 | Grassy field with fenced area | Rice husk, grass, vegetables |
|  | Zebra (*Equus zebra*) | 1 | Grassy pasture, stagnant water body, road, feeding shed | Grass, cereal grains, wheat bran |
|  | Giraffe  (*Giraffa camelopardalis*) | 2 | Grassy pasture, stagnant water body, road, feeding shed | Grass, cereal grains, wheat bran |
|  | Hippopotamus  (*Hippopotamus amphibious*) | 1 | Small grassy area, lake | Grass, cereal grains |
|  | Blackbuck  (*Antilope cervicapra*) | 1 | Grassy pasture, stagnant water body, road, feeding shed | Grass, cereal grains |
|  | Blesbuck  (*Damaliscus pygargus phillipsi*) | 1 | Grassy pasture, stagnant water body, road, feeding shed | Grass, cereal grains |
|  | Nyala (*Tragelaphus angasii*) | 1 | Grassy pasture, stagnant water body, road, feeding shed | Grass, cereal grains |
|  | Spotted deer (*Axis axis*) | 1 | Grassy pasture, stagnant water body, road, feeding shed | Grass, cereal grains, leaf |
|  | Kangaroo (*Macropus rufus*) | 1 | Grassy pasture, stagnant water body, road, feeding shed | Grass, cereal grains, leaf |
|  | Wildebeest  (Connochaetes *taurinus*) | 2 | Grassy pasture, stagnant water body, road, feeding shed | Grass, cereal grains |
|  | Gayal (*Bos frontalis*) | 2 | Grassy pasture, stagnant water body, road, feeding shed | Grass, cereal grains |
| **Carnivores** | | | | |
|  | Tiger (*Panthera tigris tigris*) | 5 | Both open natural grassland and closed enclosure | Beef/rabbit |
|  | Lion (*Panthera leo*) | 2 | Both open natural grassland and closed enclosure | Beef/rabbit |
| **Omnivores** | | | | |
|  | Asiatic Black Bear  (*Ursus thibetanus*) | 2 | Both open natural grassland and closed enclosure | Honey, Bread, Seasonal vegetables, fruits |
