# Supplementary material for: Prevalence of gastrointestinal parasitic infections in wild mammals of a safari park and a zoo in Bangladesh
Source: Vet Med Sci. 2023 Feb 6;9(3):1385–94. doi: 10.1002/vms3.1093 (PMC10188080; doi:10.1002/vms3.1093)
Supplement: Supplementary file 2 — Table S2 [file VMS3-9-1385-s002.docx]

**Supplementary table 2:** **Sample collection from animal species of CTG zoo with their habitat types and major feeding ingredients**

| **Sl. No.** | **Animal species** | **Number of Samples** | **Nature of enclosures** | **Major feed ingredients** |
| --- | --- | --- | --- | --- |
| **Herbivores** | | | | |
| 1. | Barking Deer  (*Muntiacus muntjak*) | 3 | Open-top enclosure, sandy ground with trees and vegetation, cement made shed as feeding and resting place | Grass, wheat bran, cucumber, guard, gram, carrot |
| 2. | Spotted Deer (*Axis axis*) | 4 | Open-top enclosure, sandy ground with trees and vegetation, cement made shed as feeding and resting place | Grass, cereal grain, cucumber, guard, gram, wheat bran, carrot |
| 3. | Zebra (*Equus zebra*) | 4 | Open-top enclosure, sandy ground with trees and vegetation, cement made shed as feeding and resting place | Grass, cereal grain, guard, wheat bran, gram |
| 4. | Gayal (*Bos frontalis*) | 2 | Open-top enclosure, sandy ground with vegetation, cement made shed as feeding and resting place | Grass, cereal grain, cucumber, guard, wheat bran, gram |
| 5. | Horse  (*Equus ferus caballus*) | 6 | Open-top enclosure, sandy ground with vegetation, cement made shed as feeding and resting place | Grass, cereal grain, cucumber, guard, gram, wheat bran |
| 6. | Indian crested Porcupine  (*Hystrix indica*) | 4 | Closed enclosure with cemented-floor along with water trough | Potato, guard, carrot, cucumber |
| **Carnivores** | | | | |
| 7. | Tiger  (*Panthera tigris tigris*) | 1 | Closed enclosure, cemented-floor with partial sandy ground and water trough | Beef /chicken |
| 8. | Jungle Cat (*Felis chaus*) | 1 | Closed enclosure, tiled floor along with hiding space and water trough | Beef /chicken |
| 9. | Leopard Cat  (*Proinailurus bengalensis*) | 3 | Closed enclosure , tiled floor along with hiding space and water trough | Beef /chicken |
| 10. | Fishing Cat  (*Proinailurus viverrrinus*) | 1 | Closed enclosure , tiled floor along with hiding space and water trough | Beef /chicken |
| **Omnivores** | | | | |
| 11. | Bengal Fox  (*Vulpes bengalensis*) | 2 | Closed enclosure, tiled floor, wooden box and water trough | Beef /chicken |
| 12. | Large Indian Civet  (*Viverra zebetha*) | 2 | Closed enclosure, tiled floor, wooden box for hiding and water trough | Beef/chicken, banana, eggs |
| 13**.** | Asian Palm Civet  (*Paradoxurus hermaphrodites*) | 2 | Closed enclosure, tiled floor, wooden box for hiding and water trough | Beef/chicken, banana, eggs |
| 14. | Asiatic Black Bear  (*Ursus thibetanus*) | 2 | Open-top enclosure, cemented-floor with, wood log | Gourd, rice, pumpkin, papaya, carrot, cucumber, honey, nut, broken maize |
| 15. | Rhesus Macaque  (*Macaca mulata*) | 5 | Closed enclosure, tiled floor with water trough | Bread, banana, cucumber, rice, gram, coconut, eggs, carrot |
| 16. | Gibbon (*Hoolock hoolock*) | 1 | Closed enclosure , tiled floor with water trough | Bread, banana, cucumber, coconut |
