# Supplementary material for: Prevalence of gastrointestinal parasitic infections in wild mammals of a safari park and a zoo in Bangladesh
Source: Vet Med Sci. 2023 Feb 6;9(3):1385–94. doi: 10.1002/vms3.1093 (PMC10188080; doi:10.1002/vms3.1093)
Supplement: Supplementary file 3 — Table S3 [file VMS3-9-1385-s004.docx]

**Supplementary table 3: Gastrointestinal helminth and protozoan infections with their intensity in wild mammals of BSM safari park, Bangladesh**

| **Name of animal species** | **Samples (positive)** | **Occurrence**  **(%)** | **Parasite species/genera (Occurrence %)** | **EPG/OPG**  **(Range)** | **Mean ±SEM** |
| --- | --- | --- | --- | --- | --- |
| **Herbivores** | | | | | |
| Gayal (*Bos frontalis*) | 2(2) | 100 | *Fasciola* spp. (100)  *Paramphistomum* spp. (50) | 100-200 | 338.89  ±  403.13 |
| Zebra (*Equus zebra*) | 1(1) | 100 | *Strongylus* spp. (100)  *Fasciola* spp. (100) | 200 |  |
| Wildebeest  (*Connochaetes taurinus*) | 2(2) | 100 | *Strongyloides* spp. (100)  *Fasciola* spp. (100)  *Dicrocoelium* spp. (50)  *Schistosoma* spp. (50)  *Taenia* spp. (50) | 400-800 |  |
| Blackbuck  (*Antilope cervicapra*) | 1(1) | 100 | *Strongyloides* spp. (100) | 200 |  |
| Blesbuck  (*Damaliscus pygargus phillipsi*) | 1(1) | 100 | *Strongyloides* spp. (100)  *Paramphistomum* spp. (100) | 1800 |  |
| Nyala (*Tragelaphus angasii*) | 1(1) | 100 | *Fasciola* spp. (100)  *Schistosoma* spp. (100) | 200 |  |
| Spotted deer (*Axis axis*) | 1(1) | 100 | *Fasciola* spp. (100) | 100 |  |
| Kangaroo (*Macropus rufus*) | 1(1) | 100 | *Fasciola* spp. (100) | 100 |  |
| Pony (*Equus ferus caballus*) | 2(2) | 100 | *Stongylus* spp. (100) | 200-300 |  |
| Giraffe  (*Giraffa camelopardalis*) | 2(2) | 100 | *Strongylus* spp. (50)  *Trichotongylus* spp. (100)  *Oesophagostomum* spp. (50) | 100-400 |  |
| Elephant (*Elephas maximus*) | 5(4) | 80 | *Strongylus* spp. (80) | 100-400 |  |
| Hippopotamus  (*Hippopotamus amphibious*) | 1(0) | 0 | Uninfected | 0 |  |
| **Carnivores** | | | | | |
| Lion (*Panthera leo)* | 2(2) | 100 | *Toxocara cati* (100)  *Toxascaris leolina* (50)  *Spirometra* spp. (50)  *Isospora* spp. (100) | 6800-100000 | 35666.67±  55811.95 |
| Tiger (*Panthera tigris*) | 5(1) | 20 | *Isospora* spp. (20) | 200 |  |
| **Omnivores** | | | | | |
| Asiatic Black Bear  (*Ursus thibetanus*) | 2(0) | 0 | Uninfected | 0 | 0 |
| **Total (all animals)** | **29 (21)** | **60.46** |  |  |  |
