# Supplementary material for: Prevalence of gastrointestinal parasitic infections in wild mammals of a safari park and a zoo in Bangladesh
Source: Vet Med Sci. 2023 Feb 6;9(3):1385–94. doi: 10.1002/vms3.1093 (PMC10188080; doi:10.1002/vms3.1093)
Supplement: Supplementary file 4 — Table S4 [file VMS3-9-1385-s001.docx]

**Supplementary table 4: Gastrointestinal helminth and protozoan infections with their intensity in wild mammals of CTG zoo, Bangladesh**

| **Name of animal species** | **Samples**  **(Positive)** | **Occurrence**  **(%)** | **Parasite species/genera**  **(Occurrence %)** | **EPG/OPG**  **(Range)** | **Mean**  ±  **SEM** |
| --- | --- | --- | --- | --- | --- |
| **Herbivores** | | | | | |
| Indian crested Porcupine  (*Hystrix indica*) | 4(3) | 75 | *Eimeria* spp. (100)  *Trichuris* spp. (50) | 200-85600 | 6466.67  ±  21982.68 |
| Gayal (*Bos frontalis*) | 2(2) | 100 | *Eimeria* spp. (100) | 100 |  |
| Horse (*Equus ferus caballus*) | 6(6) | 100 | *Strongylus* spp*.* (100) | 200-600 |  |
| Zebra (*Equus zebra*) | 4(4) | 100 | *Strongylus* spp*.* (100)  *Strongyloides* spp. (25)  *Triodontophorus* spp. (25) | 100-400 |  |
| Spotted Deer (*Axis axis*) | 3(0) | 0 | Uninfected | 0 |  |
| Barking Deer  (*Muntiacus muntjak*) | 3(0) | 0 | Uninfected | 0 |  |
| **Carnivores** | | | | | |
| Fishing Cat  (*Prionailurus viverrinus*) | 3(1) | 33.33 | *Toxocara cati* (33.33) | 800 | 7875  ±  14751.58 |
| Jungle Cat (*Felis chaus*) | 1(1) | 100 | *Toxocara cati* (100)  *Toxascaris leolina* (100)  *Strongyloides* spp*.* (100)  *Spirometra* spp. (100) | 30000 |  |
| Lion (*Panthera leo*) | 1(1) | 100 | *Toxocara cati* (100)  *Toxascaris leolina* (100) | 300 |  |
| Leopard Cat  (*Prionailurus bengalensis*) | 1(1) | 100 | *Ancylostoma* spp. (100)  *Spirometra* spp. (100) | 400 |  |
| Tiger (*Panthera tigris tigris*) | 1(0) | 0 | Uninfected | 0 |  |
| **Omnivores** | | | | | |
| Bengal Fox (*Vulpes bengalensis*) | 2(2) | 100 | *Spirometra* spp. (100) | 800-4000 | 1428.57  ±  1642.88 |
| Asiatic Black Bear  (*Ursus thibetanus*) | 2(0) | 0 | Uninfected | 0 |  |
| Large Indian Civet  (*Viverra zibetha*) | 2(2) | 100 | *Ancylostoma* spp. (100)  *Strongyloides* spp. (100) | 1500-3400 |  |
| Asian Palm Civet  (*Paradoxurus hermaphroditus*) | 2(0) | 0 | Uninfected | 0 |  |
| Gibbon (*Hoolock hoolock*) | 1(1) | 100 | *Ancylostoma* spp. (100) | 100 |  |
| Rhesus Macaque  (*Macaca mulatta*) | 5(2) | 40 | *Ancylostoma* spp. (40) | 100 |  |
| **Total (all animals)** | **43 (26)** | **72.41** |  |  |  |

EPG/OPG = Egg Per Grams/Oocyst Per Gram, SEM = Stadard Error of the Mean
